# Real world data in health technology assessments in kidney transplants in Germany: use of routinely collected data to address epidemiologic questions in kidney transplants in the AMNOG process in Germany

**DOI:** 10.3205/000263

**Published:** 2018-01-24

**Authors:** Kirsten H. Herrmann, Ulf Meier-Kriesche, Aljoscha S. Neubauer

**Affiliations:** 1 Bristol-Myers Squibb, Munich, Germany; 2 Bristol-Myers Squibb, Lawrenceville, US; 3 Institute for Health Economics (IfG), Munich, Germany

**Keywords:** kidney transplantation, kidney transplant, epidemiology, incidence, prevalence, Germany

## Abstract

**Introduction:** It is discussed whether real world data can be used in health technology assessment. Following it is of interest whether routinely collected data for quality assurance (QA) in the hospital sector is feasible to address epidemiologic questions in kidney transplantation in the AMNOG process in Germany.

**Objectives:** To investigate the proportion of kidney transplants classified as from so-called standard criteria donors (SCD) and from expanded criteria donors (ECD) in Germany and to study the age distribution.

**Methods:** After granted use by the Federal Joint Committee (G-BA), the data analysis was carried out by the AQUA institute, and a SPSS code was developed. Special challenge was the complex definition of SCD/ECD criteria that, in addition to donor age, takes into account combinations of donor diagnoses, creatinine, and cold ischemia time.

**Results:** Age analyses could be performed in all patients. Median age of the adult transplant recipients in Germany was 54 years in 2012 as well as in 2013, range 18–85 and 18–82 years and a mean (SD) of 53 (14) and 52 (14) years, respectively. 63.5% (2012) and 62.5% (2013) of recipients were male. Classification in SCD/ECD transplants could be performed for 2,083 of 2,461 patients (85%; 2012) and for 1,795 of 2,079 patients (86%; 2013). Of all classifiable transplants 61.4% (2012) and 66.5% (2013) were SCD transplants. Total project time from the request to results was <6 months.

**Conclusions:** The use of data routinely collected for QA in the hospital sector is feasible to address epidemiologic questions in kidney transplantation in the AMNOG process in Germany, which is basically following the systematic of an HTA process. All patients with kidney transplants are represented thus avoiding sampling error. Limitations include the availability of all necessary data in the QA data set. Within <6 months’ time with reasonable resources it was possible to meet timelines. The analyses were accepted by the authorities.

## Zusammenfassung

**Hintergrund:** Es wird diskutiert, ob Routinedaten im Rahmen von Health Technology Assessments (HTA) genutzt werden können. Zur Beantwortung von epidemiologischen Fragestellungen zur Nierentransplantation in Deutschland im AMNOG Verfahren sollten Qualitätssicherungsdaten nach §137a SGB V genutzt werden.

**Ziele:** Die Altersverteilung von Spendern und erwachsenen Empfängern für die Nierentransplantation in Deutschland sollte möglichst exakt ermittelt werden. Zusätzlich sollte der Anteil von Transplantationen nach den sogenannten Standardkriterien (SCD) und erweiterten Kriterien (ECD) bestimmt werden.

**Methoden:** Nach positiver Beantwortung des formalen Antragsverfahrens durch den gemeinsamen Bundesausschuss (G-BA) erfolgte die Datenanalyse durch das AQUA-Institut basierend auf einer SPSS-Abfrage. Eine besondere Herausforderung hierbei war die komplexe Definition der sogenannten Standardkriterien (SCD) und erweiterten Kriterien (ECD), die neben Spenderalter auch Kombinationen von Spenderdiagnosen sowie dem Laborwert Kreatinin sowie die kalte Ischämiezeit berücksichtigt.

**Ergebnisse:** Für die Altersanalysen ließen sich alle Patienten klassifizieren. Das mediane Alter der erwachsenen Transplantatempfänger in Deutschland betrug im Jahr 2012 und Jahr 2013 jeweils 54 Jahre, bei einer Spannweite von 18–85 bzw. 18–82 Jahren und einem Mittelwert (Standardabweichung) von 53 (14) bzw. 52 (14) Jahren. 63,5% (2012) bzw. 62,5% (2013) der Empfänger waren Männer. Die komplexe Klassifikation in SCD/ECD Transplantate war bei 2.083 von 2.461 Patienten (85%) im Jahr 2012 bzw. 1.795 von 2.079 Patienten (86%) im Jahr 2013 möglich. Es ergab sich ein Anteil von SCD-Transplantationen an allen klassifizierbaren Transplantaten von 61,4% im Jahr 2012 und 66,5% im Jahr 2013. Die Gesamtlaufzeit des Projektes vom Tag der Beantragung bis zum finalen Ergebnis betrug unter 6 Monaten.

**Fazit:** Die Nutzung der im Rahmen der externen stationären Qualitätssicherung gem. §137a SGB V erhobenen Daten für epidemiologische Fragestellungen im AMNOG-Verfahren ist möglich. Im Bereich Nierentransplantation besteht eine Vollerhebung für Deutschland, so dass Stichprobenfehler vermieden werden und qualitativ hochwertige, repräsentative Auswertungen erfolgen können. Wesentliche Limitation der Sekundärdatenanalyse ist der zur Verfügung stehende Umfang des Datensatzes, der primär auf die Zwecke der externen Qualitätssicherung ausgerichtet ist. Mit einer Projektlaufzeit <6 Monaten war es möglich, die Fristen einzuhalten und die Analysen wurden vom gemeinsamen Bundesausschuss akzeptiert.

## Introduction

Kidney transplantations are the most frequently performed transplantations of solid organs in Europe and in Germany [1]. Surgery is performed as an in-patient hospital procedure. In the health care system in Germany all in-patients after kidney transplantation are covered by the compulsory national quality assurance (QA) program according to §137a SGB V. The performed secondary analyses of the obtained data are focused on QA goals, e.g. transplant outcomes from living donors and post-mortal donors are analyzed and reported [2].

It is discussed whether real world data can be used in health technology assessment (HTA). Following an example in the German healthcare system, it is of interest whether routinely collected data for quality assurance (QA) in the hospital sector is feasible to address epidemiologic questions in kidney transplantation in the AMNOG process in Germany. In the AMNOG two substances are compared basically following the HTA process. As a result there is a recommendation based on the benefit assessment. In this process it was the objective to find out whether the population characteristics of clinical studies is representative for kidney transplant recipients in the German health care environment. 

To answer the question of age comparability, the age distribution of recipients of kidney transplants (combined living and postmortal organs) were required to be calculated as representatively as possible for Germany. The routinely published QA results, however, did not provide sufficient analysis depth. For instance, the detailed age distribution of all adult kidney transplant recipients is not available in the federal analysis reports to allow e.g. age adjustments of clinical study data. Furthermore, it is well known that the organ quality of the kidney transplants impacts survival of the transplanted organ: the literature therefore distinguishes the so-called standard criteria donors (SCD) and extended criteria donors (ECD) for many years [3]. ECD donors are frequently used in Europe due to donor organ shortage. However, exact numbers regarding the shares of SCD and ECD transplantations, were not available from the literature or federal analysis reports to representatively cover Germany. Therefore, the SCD/ECD distribution for Germany and detailed age distribution were investigated in this study based on nationwide QA data.

## Methods

A secondary analysis of QA data was planned to answer the questions regarding age distributions and ECD/SCD percentages. The QA process according to § 137a SGB is mandatory for all hospitals performing kidney transplants in Germany and therefore covers all patients with kidney transplants.

The use of QA data requires approval via a formal application process. The Federal Joint Committee (G-BA) decides after a formal application outlining the goals and the data analysis process [4]. The data analysis itself was carried out by the AQUA institute (https://www.aqua-institut.de), which has been commissioned and authorized by the G-BA pursuant to the social security code article §137a SGB V between 2009 and 2015 to collect, analyze and publish the nationwide results from the QA. From 2016 onwards the responsibility for these processes where handed over to the IQTIG institute in Berlin (https://www.iqtig.org). Necessary data privacy protection is assured by data processing of only anonymized data and in addition by presentation of the results only in aggregated form.

Based on the field description and dummy data record set, SPSS syntax was developed to answer the analysis questions. The code was optimized together with the AQUA institute in several review rounds and was finally implemented then on the 2012 and 2013 data set, which was the most recent available fully cleaned complete data in early 2015. In contrast to the usually separate QA analyses, the data from living donors and post-mortem donors were combined for all analyses, to allow a comprehensive view on all kidney transplantations in Germany. Due to the primary HTA question of age comparability in the AMNOG process and a clinical study population limited to adult recipient patients, the analysis was limited to recipients ≥18 years.

Necessary data privacy protection was assured by data processing of only anonymized data and in addition by presentation of the results only in aggregated form.

**Figure 1:**
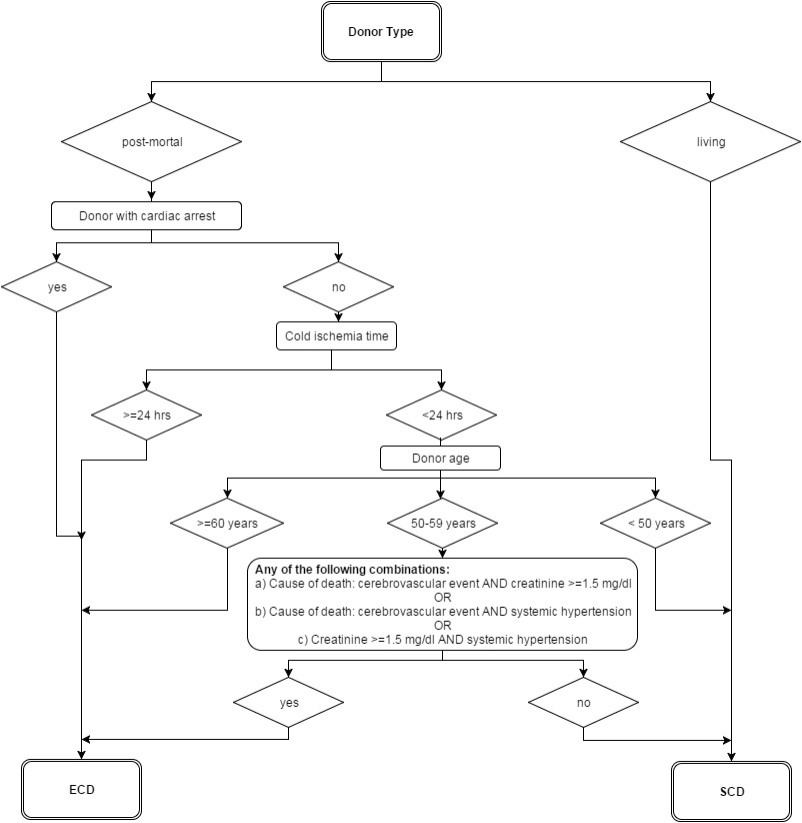
Classification of donors into standard criteria donors (SCD) and extended criteria donors (ECD)

The age analyses could be implemented by simple frequency counts after combining and selecting the appropriate datasets. In contrast, the definition of the SCD/ECD donors based on the published criteria was relatively complex: The SCD/ECD categorization [3] takes into account not only donor age but also combinations of age with donor diagnoses, the laboratory value creatinine and cold ischemia time. Figure 1 (Fig. 1) summarizes the algorithm used for definition of SCD/ECD (donation after final cardiac arrest not legally possible in Germany).

Since not all criteria were documented in the routine QA, some of the patients had to be classified as “not classifiable”, if relevant information was not available to allow certain donor grouping. This was most relevant in the 50–59 years donor group (see Figure 1 (Fig. 1)). Of note, all living donors always are considered SCD donors. The final SPSS code used for the data analysis for ECD/SCD differentiation is provided as an Appendix.

## Results

**Figure 2:**
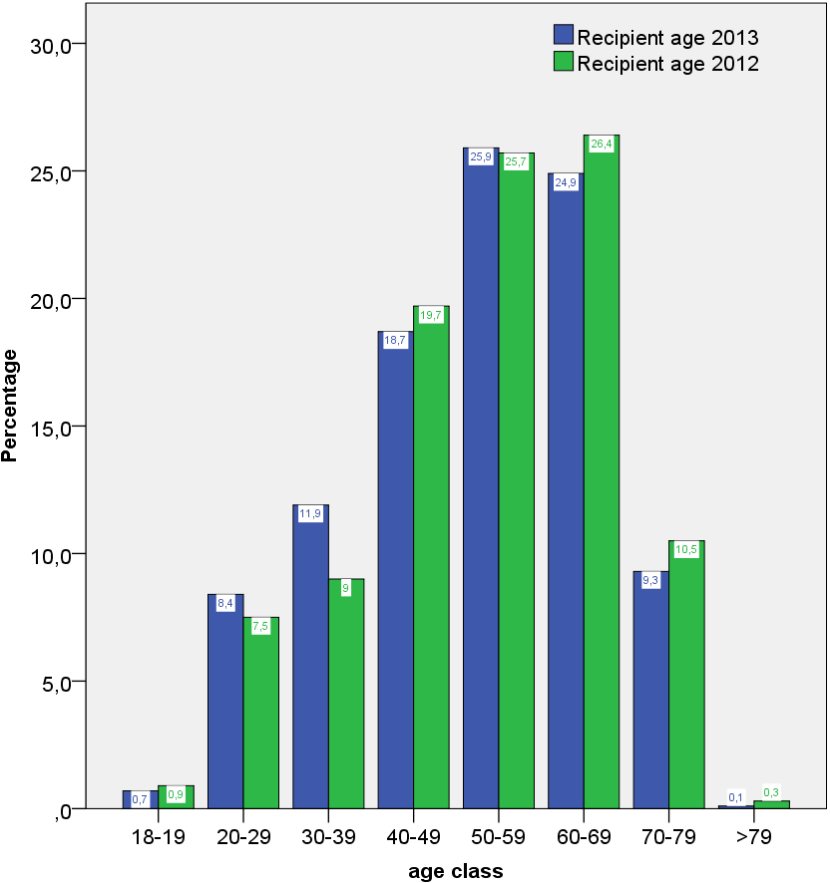
Age distribution of all adult kidney transplant recipients in 2012 and 2013

Age analyses could be performed in all recipients documented in the QA data. In 2012, 2,556 kidney transplantations were documented, while 2,163 transplantations were documented in 2013. Of these, adult recipients were 2,461 patients in the year 2012 and 2,079 in the year 2013. The median age of the adult transplant recipients in Germany was 54 years in 2012 as well as in 2013, with a span of 18–85 and 18–82 years, respectively. The mean (standard deviation) was 53 (14) years in 2012 and 52 (14) years in 2013 (Figure 2 (Fig. 2)).

In 2012 a total of 63.5% and in 2013 62.5% of transplant recipients were male. Regarding donors, a total of 30.6% in 2012 and of 33.7% in 2013 were living kidney donors.

**Figure 3:**
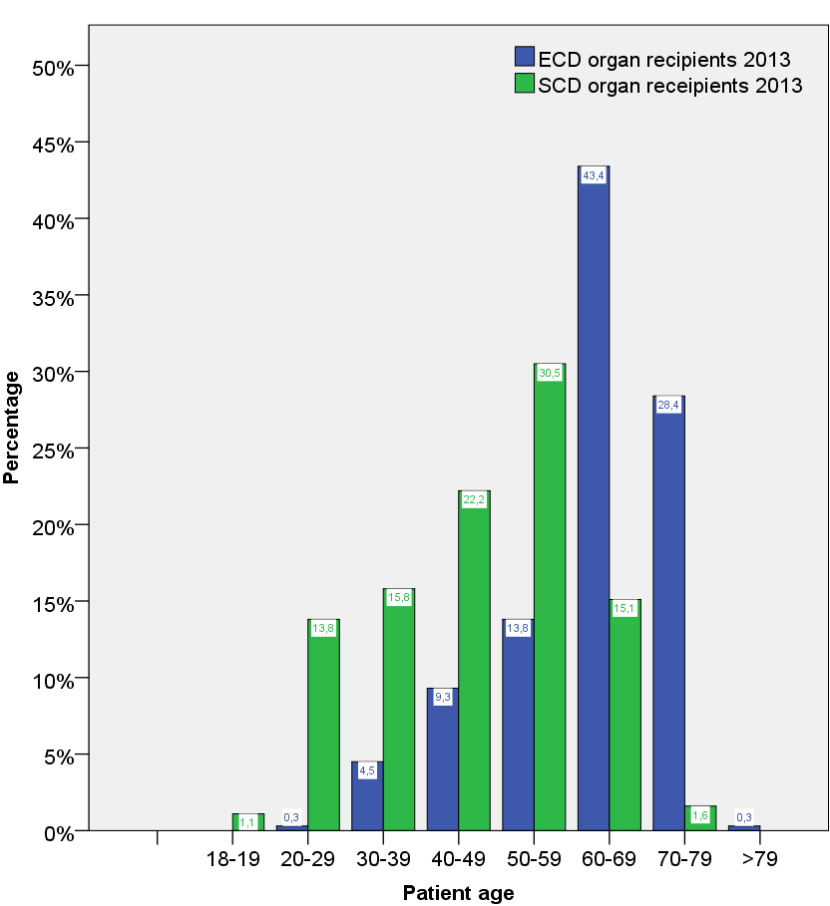
Age distribution of all kidney transplant donors (living and post-mortem) in 2012 and 2013

Of all donors, age information was available in 95.9% in 2012 and 94.9% in 2013. Figure 3 (Fig. 3) summarizes valid percentage distribution of all kidney transplant donors, including post-mortem and living donors.

In 2012 a total of 48.8% and in 2013, 51.1% of all transplant donors were female.

The complex classification in SCD/ECD transplants could be performed for 2,083 of 2,461 adult patients (85%) in 2012 and for 1,795 of 2,079 adult recipients (86%) in 2013. It resulted in a share of 52.0% SCD transplants in 2012 and 57.4% SCD transplants in 2013 (Table 1 (Tab. 1)).

**Figure 4:**
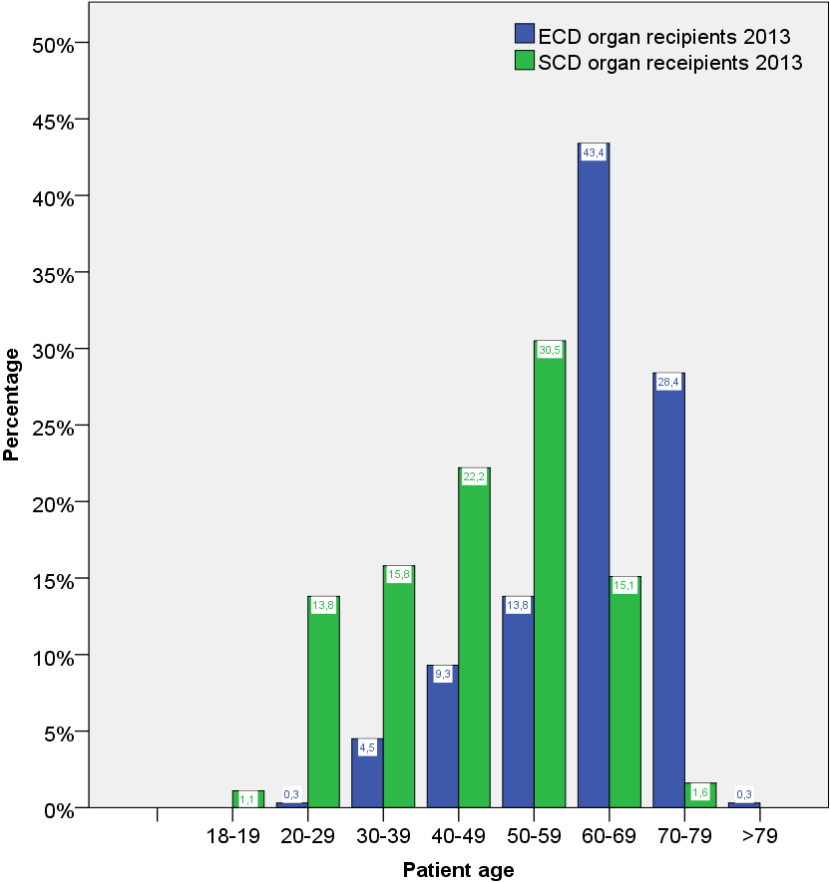
Age distribution of all adult kidney transplant recipients (living and post-mortem) with ECD and SCD classified organs in 2013

Age distribution of SCD and ECD organ recipients differed clearly, which is shown for 2013 in Figure 4 (Fig. 4). The patients receiving ECD organs were clearly older than those receiving organs classified as SCD.

The total project time from filing the request for data analysis to the G-BA till the final result was less than 6 months. Further clinically relevant questions after kidney transplantation such as Epstein-Barr virus (EBV) status or immunosuppression medication regimen could not be answered from the routine QA data due to missing recording of those data.

## Discussion

The current study for the first time investigated a complete data set of all yearly German living and post-mortal kidney transplantations to describe important epidemiology data. In the AMNOG process (HTA like process) routine data were accepted by G-BA [5] to demonstrate, population characteristics respective the age of the study population would mirror the characteristics of kidney transplant recipients in the German health care environment. The data could show age and gender distributions for donors and adult recipients and classify ECD and SCD organs. Strengths of the study are the nearly full coverage of all patients and donors and recent data: in 2013 of expected renal transplant data sets 99.8% were obtained and in 2012 also 99.8% of expected transplant data sets were obtained [2]. At the same time, data quality underlying the study can be considered high due to using QA data. This data were supplied by the transplanting hospitals based on their patient charts. A formal data validation process was in place: it consisted of comparing expected data sets vs. received data sets and various checks for plausibility (e.g. duplicates and inconsistencies). In addition, a random sample of hospitals was selected each year for several data fields and the data were checked for those random samples [6]. For the classification of ECD and SCD organs approximately 15% of the donors (Table 1 (Tab. 1)) could not be classified based on the available QS dataset: This was due to data missing in the definition of the QS dataset, which would have been necessary for the classification. This limitation mainly is based on non-existence of information on systemic hypertension of the donor and non-inclusion of cold ischemia time in the QA data set after the data year 2012.

Preferably it would have been possible to investigate more clinically relevant epidemiologic data such as immunosuppression patterns or EBV status of kidney transplantation patients. However, one well known limitation with secondary analysis is that the data is primarily collected for different purpose – here the QA – which limits their applicability.

Compared with the routine publications by the AQUA institute for the years investigated [2], some underreporting of total number of cases was observed in the analysis. This is due to hospitals failing the requirement as well as so-called “Überlieger”, i.e., patients who stay in hospital over the course of the calendar years. Such missing data from the hospitals may be obtained after the respective data delivery time points and leads to slight corrections of numbers in the subsequent QS report. Another limitation of the data is that the quality assurance data focuses on the recipient patients, i.e., the transplantation and not on donors: Thus, an individual donor donating two kidneys to two recipient patients is counted “double” for both transplantations. This needs to be considered when assessing the data.

Compared with other approaches, for instance for the SCD percentage, the literature gives 72.3% SCD for Germany [7], published in 2009 based on patients randomized in a clinical trial in the years 2002 to 2004. The Institute for Quality and Efficiency in Healthcare (IQWiG) calculated 63% SCD for Germany based on a simple age cut of 55 years [8]. This was resulting from a scarcity of recent publications. The IQWIG result is well in line with the data obtained in our current study based on a detailed analysis of QA data.

Other data sources include the number of transplanted organs from the organizations Eurotransplant and the German Organ Transplantation Foundation (Deutsche Stiftung Organtransplantation DSO). Summary data is regularly published on the national German healthcare data website (https://www.gbe-bund.de). For the year 2012 a total of 2,586 transplantations and for 2013 in total 2,272 transplantations are listed, as compared to 2,570 and 2,262 transplantations covered by the QA data [2]. This confirms completeness of QA data, while the transplant organization data gives more details on donors but is lacking recipient statistics [1]. Discrepancy to included recipient patient number in our analysis (Table 1 (Tab. 1)) is mainly due to not considering recipients <18 years of age (see age distribution in [2]) and to lesser degree based on QA data sets not fully delivered and/or validated. 

To sum up: The use of data routinely collected for QA in the hospital sector is feasible to address epidemiologic questions in kidney transplantation in the AMNOG process in Germany, which is basically following the systematic of an HTA process. All patients with kidney transplants are represented thus, avoiding sampling error. Limitations include the availability of all necessary data in the QA data set. Within <6 months’ time with reasonable resources it was possible to meet timelines. All patients in the field of kidney transplantation in the German in-patient sector are represented in this data set. Thus, sampling error can be avoided and in consequence high-quality, representative evaluations are obtained. The analysis results from this study were accepted by the authorities (the Federal Joint Committee, G-BA) in the AMNOG process to demonstrate, population characteristics respective the age of the study population would mirror the characteristics of kidney transplant recipients in the German health care environment.

## Appendix

### SPSS code for ECD/SCD differentiation

COMPUTE catspen=0.

VARIABLE LABELS catspen 'Spenderkategorie'.

VALUE LABELS catspen 0 'unbekannt' 1 'ECD' 2 'SCD'.

DO IF ALTERSPEN>49 AND ALTERSPEN <60.

IF (TodesursacheSpen=218 OR TodesursacheSpen=229 OR TodesursacheSpen=230

OR TodesursacheSpen=234 OR TodesursacheSpen=235 OR TodesursacheSpen=236)

AND KREATININWERTSPMGDL>=1.5 catspen=1.

IF (TodesursacheSpen~=218 AND TodesursacheSpen~=229 AND TodesursacheSpen~=230 AND TodesursacheSpen~=234 AND TodesursacheSpen~=235 AND TodesursacheSpen~=236)

AND KREATININWERTSPMGDL<1.5 catspen=2.

END IF.

IF ALTERSPEN>59 catspen=1.

IF ALTERSPEN<50 catspen=2.

* In data year 2013 no cold ischemia recorded, in data year 2012 following line valid.

* IF ISCHAEMIEZEITKALT>1440 catspen=1.

IF SPENDERTYP=2 catspen=2.

FREQUENCIES catspen.

## Notes

### Competing interests

Kirsten H. Herrmann, Ulf Meier-Kriesche: employees of BMS at the time of the study. Aljoscha S. Neubauer: research support from BMS.

### Contributions

Kirsten H. Herrmann designed study, performed research, wrote paper; Ulf Meier-Kriesche designed study; Aljoscha S. Neubauer performed research, wrote the paper.

### Acknowledgements

We thank Thomas König, Institute for Applied Quality Improvement and Research in Health Care (AQUA), Göttingen, for preparing and conducting the analysis. Uli Jeratsch, Advanced Medical Services (AMS), Munich, Germany, for preparing and revising the code for the analyses. Wojciech Dombrowsky, (Bristol-Myers Squibb, Munich, Germany, until 2016), Germany, for medical advice and revising the paper. 

## Figures and Tables

**Table 1:**
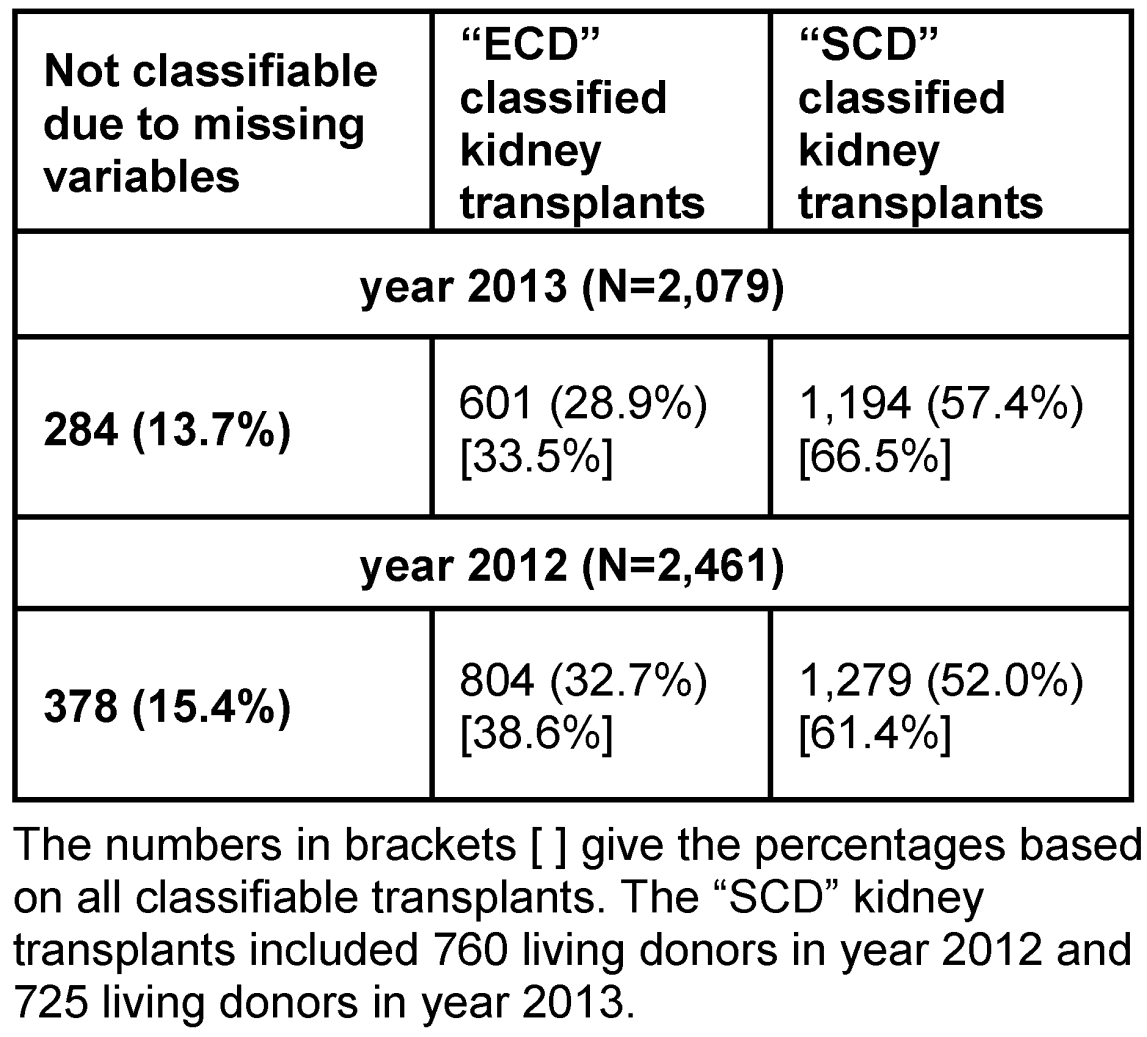
Kidney transplants classified standard criteria donors (SCD) and extended criteria donors (ECD) for year 2012 and 2013
